# Increasing the Production of β-Glucan from *Saccharomyces carlsbergensis* RU01 by Using Tannic Acid

**DOI:** 10.1007/s12010-021-03553-5

**Published:** 2021-03-31

**Authors:** Natthaporn Chotigavin, Wiramsri Sriphochanart, Surachai Yaiyen, Sanya Kudan

**Affiliations:** 1 Program in Food Science, Faculty of Food Industry, King Mongkut’s Institute of Technology Ladkrabang, Bangkok, 10520 Thailand; 2 Program in Fermentation Technology in Food Industry, Faculty of Food Industry, King Mongkut’s Institute of Technology Ladkrabang, Bangkok, 10520 Thailand; 3 Department of Art and Science Technology, Western University, Lumlukka, Pathumthani Province 10350 Thailand; 4 Department of Biotechnology, Faculty of Science, Ramkhamhaeng University, Bangkok, 10240 Thailand

**Keywords:** Cultivation mode, β-Glucan, Molasses diammonium sulfate (MDS) medium, *Saccharomyces carlsbergensis* RU01, Stirred tank reactor (STR), Tannic acid (TA)

## Abstract

In this study, we increased β-glucan production from brewer’s yeast, *Saccharomyces carlsbergensis* RU01, by using tannic acid. High-pressure freezing and transmission electron microscopy (HPF-TEM) revealed that the yeast cell wall obtained from yeast malt (YM) medium supplemented with 0.1% w/v tannic acid was thicker than that of yeast cultured in YM medium alone. The production of β-glucan from *S. carlsbergensis* RU01 was optimized in 3% w/v molasses and 0.1% w/v diammonium sulfate (MDS) medium supplemented with 0.1% w/v tannic acid. The results showed that MDS medium supplemented with 0.1% w/v tannic acid significantly increased the dry cell weight (DCW), and the β-glucan production was 0.28±0.01% w/v and 11.99±0.04% w/w. Tannic acid enhanced the β-glucan content by up to 42.23%. β-Glucan production in the stirred tank reactor (STR) was 1.4-fold higher than that in the shake flask (SF) culture. Analysis of the β-glucan composition by Fourier transform infrared (FTIR) spectroscopy showed that the β-glucan of *S. carlsbergensis* RU01 cultured in MDS medium supplemented with 0.1% w/v tannic acid had a higher proportion of polysaccharide than that of the control. In addition, β-glucans from brewer’s yeast can be used as prebiotic and functional foods for human health and in animal feed.

## Introduction

β-Glucans are polysaccharide molecules composed of glucose units and can be found in bacteria, algae, yeasts, mushrooms, molds, and higher plants. Their structure depends on the source of the β-1,3 linkage, and each such molecule possesses many novel properties and can improve human and animal health and the immune system. β-1,3-Glucans are classified as biological response modifiers. The molecular mass, shape, structure, and source of β-glucans that provide the most significant therapeutic benefit are highly diverse [1]. According to a previous report, the most bioactive β-glucans contain 1,6-linked sidechains branching off from the more extended β-1,3-glucan backbone and are referred to as β-1,3/1,6-glucans [2]. Many reports have suggested the production of β-glucans from different sources, such as fungi, bacteria, algae, oats, and barley [3–8], which show different linkage types, molecular weights, and degrees of branching [9, 10]. One of the sources of β-glucan is the yeast *Saccharomyces cerevisiae* cell wall, which is composed of approximately 55–65% β-glucan [11]. Kim and Yun [12] and Liu et al. [13] studied the technique of β-glucan production and isolation from *S. cerevisiae*. They achieved high productivity and purity with clean and mild treatment of β-glucan from the yeast cell wall. A method has been developed to produce a high level of yeast glucan from agricultural waste such as molasses and corn steep liquor (CSL) with fed-batch culture fermentation [14].

Tannins are water-soluble polyphenols found in plant species used in the brewing industry, e.g., grape, malts, and hops, while mashing the boiling wort [15]. Tannins are known to inhibit yeast growth and metabolism in the fermentation process [16]. For this reason, many reports have studied the interaction between wine tannins and yeast cells [17, 18]. It was suggested that yeast could fix tannins located in the cell wall. When tannins interact with the yeast cell wall, proteins and polysaccharides in the cell wall are precipitated [19]. According to the interaction, colloidal aggregates can form, and these aggregates have a limited size and remain stable. The result is the prevention of glycosyl moieties forming multiple bridges between tannins and their protein parts. Subsequently, hydrophilic and negatively charged aggregates are formed. This process stops yeast cell growth [19, 20]. In addition, it has been suggested that under tannin stress conditions, yeast can protect and maintain internal homeostasis using β-glucan accumulation in the cell wall. According to microscopic observations, the yeast cells appeared thicker [17]. Conventionally, brewer’s yeast strains are divided into two categories, namely, top-fermenting (ale) and bottom-fermenting (lager) yeasts. Strains of *S. cerevisiae* are commonly used to produce ales in the temperature range of 16–25 °C. On the other hand, *Saccharomyces carlsbergensis* strains are industrially used to produce lagers in the temperature range from 8 to 15 °C [21]. The yeast *S. carlsbergensis* is commonly used for beer production. The waste production from this beer fermentation process is high. *S. carlsbergensis*, however, is rich in protein, vitamin B, chitin, and β-glucans, which possess several physiological functions. Interestingly, breweries can generate additional revenue by isolating β-glucan from spent beer yeast as a high-value product. Tian et al. [22] used alkali treatment at high pressure to isolate β-glucan from spent beer yeast. An extraction rate of 78.38% with 78.11% β-D-glucan content was achieved under optimal conditions. Homogenization of cell walls was found to increase the yield of β-glucan [23, 24]. Alkaline treatment was used to isolate β-glucan from the cell walls of spent brewer’s yeast; β-glucan with minimal structural changes could be obtained by combination of sonication and spray-drying, and the formed particles exhibited an insignificant amount of agglomerate formation [24]. However, these properties of β-glucans were shown to be affected by differences in isolation and drying procedures. They found that lyophilized preparations exhibited the highest oil-binding capacity and lowest swelling, and air-dried preparations showed enhanced swelling. It has also been shown that β-glucans obtained from yeast homogenized cell walls exhibit relatively high apparent viscosity, emulsion-stabilizing capacity, and water-holding capacity than commercial β-glucans from baker’s yeast [23]. A benefit of β-glucans is also its ability to act as a potent stimulator of the immune system against infection by viruses, bacteria, and fungi, which leads to cancer and stress-related immune suppression [25–27].

This study aimed to investigate the effect of tannic acid on cell morphology, dry cell weight (DCW), and β-glucan production in *S. carlsbergensis* RU01. The cultivation mode of β-glucan production in molasses and diammonium sulfate (MDS) medium supplemented with tannic acid was studied.

## Materials and Methods

### Yeast Strain

*S. carlsbergensis* strain RU01 was isolated from beer beverage waste. For long-term maintenance, the strain was stored at −20 °C in yeast malt (YM; Himedia) medium supplemented with 0.1% w/v tannic acid (Sigma) and 20% w/v glycerol (Sigma). Before the experiment, the strain was propagated twice in YM medium supplemented with 0.1% w/v tannic acid.

### Transmission Electron Microscopy (TEM) of the Yeast Cell Wall

*S. carlsbergensis* RU01 was available as a cryofixed (by the high-pressure freezing (HPF) method) unit that exhibits reliable physical performance in yeast cells [28]. The cells of *S. carlsbergensis* strain RU01 were grown in YM supplemented with 0.1% w/v tannic acid and in YM as a control at 30 °C for 48 h. Some of the cultures were starved in a refrigerator at 4 °C, and some were suspended in 20% v/v glycerol for at least 4 h. *S. carlsbergensis* RU01 was cryofixed by HPF using an HPM 010 (BAL-TECAG, Liechtenstein) at −193 °C and 210 MPa. The cells were observed after cryocutting at −175 to −185 °C. Yeast cell sections with a thickness of 100 nm were prepared. The samples were imaged with a Philips CM10 transmission electron microscope. The structures of yeast in near-native state were studied.

### Yeast Cultivation Mode

#### Shake Flask **(**SF**)** Culture

*S. carlsbergensis* RU01 was cultured in 3% w/v molasses and 0.1% w/v diammonium sulfate (MDS) medium supplemented with 150 mL of 0.1% w/v tannic acid in a 250-mL Erlenmeyer flask at 30 °C, pH 5.0, and 200 rpm for 36 h. The growth rate was measured at 600 nm. The dry weight was determined. Then, 2 mL of culture was diluted to 50 mL, filtered through a predried filter (0.45 μm pore size), and washed twice with 50 mL of normal saline solution. Cells on the filters were dried at 100 °C in an oven until constant weight and were subsequently cooled at room temperature in a desiccator for 2 h before weighing. The DCW was the difference between the filter weights with or without yeast.

#### Bioreactor-Scale Culture

Scale-up of the culture was performed in a 7.5-L bioreactor (BIOFLO310, New Brunswick, USA) with a 5-L working volume. *S. carlsbergensis* RU01 was cultured in MDS medium supplemented with 0.1% w/v tannic acid at 30 °C and pH 5.0 for 48 h. The medium was inoculated with an initial concentration of approximately 6.0×10^7^ CFU/mL (250 mL). Three agitation rates (100, 200, and 400 rpm) and two aeration rates (0.5 and 1.0 vvm) were used.

### β-Glucan Extraction and Determination

#### Alkaline Extraction

The cell wall of *S. carlsbergensis* RU01 was extracted by a modified alkaline extraction method [29]. The cell wall was extracted with 6% w/v NaOH at 90 °C for 2 h and centrifuged at 6000 rpm and 4 °C for 10 min; the supernatant was discarded. The sediment was washed three times in distilled water, and the supernatant was removed by centrifugation at 6000 rpm and 4 °C for 10 min. Subsequently, the pH was adjusted to 6.0–7.0 with 1% v/v HCl. The isolated material was designated β-glucan because it contained this polysaccharide as the main component, mixed with a small number of other polysaccharides.

#### Analyses of β-Glucan

According to the manufacturer’s instructions, the 1,3/1,6-β-glucans were determined in quadruplicate using an assay kit [30]. The β-glucan content was determined by subtracting the α-glucan content from the total glucan content. The total glucan/α-glucan levels and the D-glucose in the oligosaccharide, sucrose, and free-D-glucose contents were measured in both steps. The enzymatic assay test for detecting 1,3/1,6-β-glucans in yeast is a complete method for quantitative determination of specific linked β-glucans in yeast. All glucans were split into their glucose monomers and measured spectrophotometrically.

#### Fourier Transform Infrared **(**FTIR**)** Spectroscopic Analysis

Powdered glucan was analyzed by FTIR spectroscopy (Bruker Invenio S instrument, UK). FTIR spectra were obtained in absorption mode at room temperature. Spectra were recorded from 400 to 4000 cm^−1^. Spectra were preprocessed with OPUS-TOUCH software. The curve fitting used to quantify the ratio of polysaccharides, proteins, and lipids was based on a least-square method using Gaussian bands.

### Statistical Analysis

All experiments were performed in triplicate, and analysis of variance (ANOVA) with a confidence interval of 95% (*p*<0.05) was reported. The significance of the results was validated using SPSS (version 18.0).

## Results and Discussion

### Cell Wall Remodeling Accompanies Cell Volume Changes During Tannic Acid Stress

**Fig. 1:**
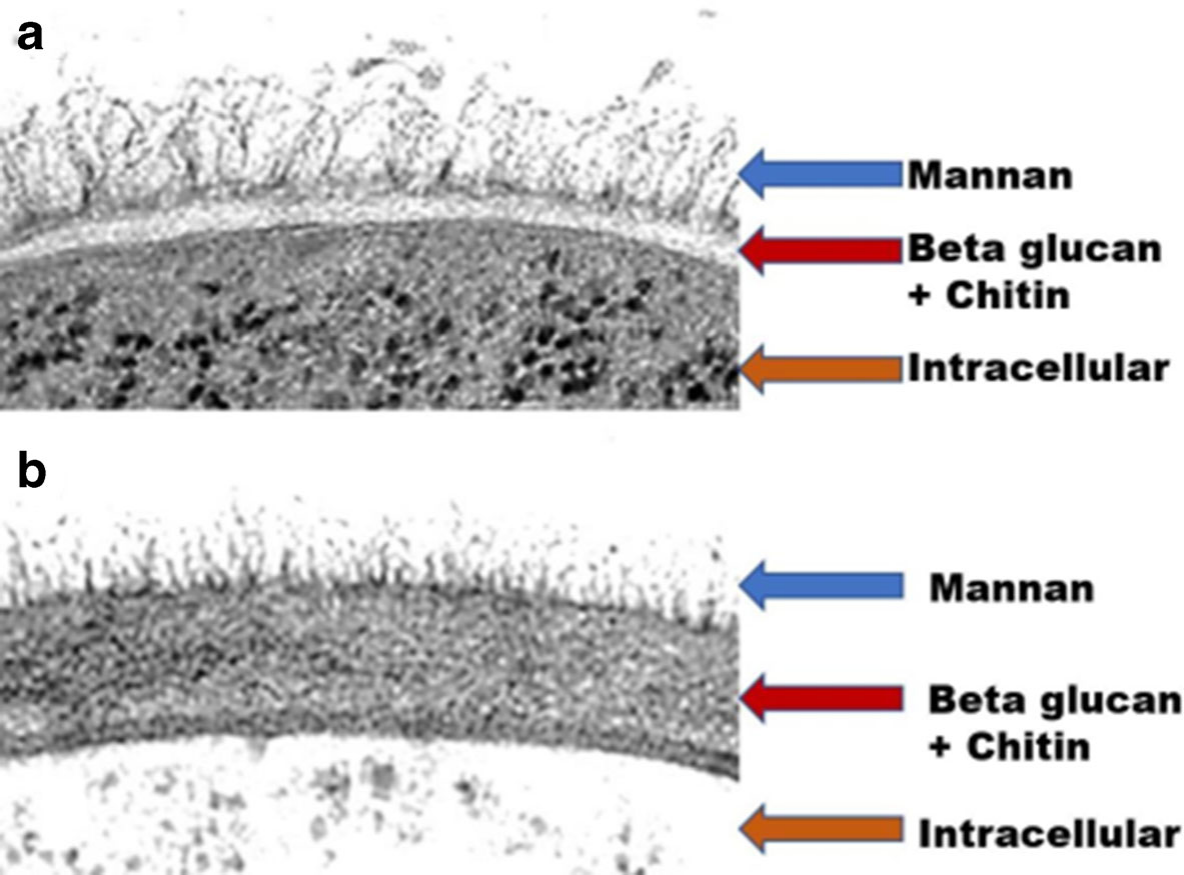
For transmission electron microscopy (TEM) of the *Saccharomyces carlsbergensis* RU01 cell wall, the cells were fixed with high-pressure freezing (HPF) after cultivation in YM medium without (**a**) or with (**b**) 0.1% w/v tannic acid supplementation

In a preliminary study, it was found that MDS medium interfered with the HPF technique. In this report, YM medium was used instead of MDS medium to study the cell wall structure. Moreover, YM medium is consistently applied in large-scale fermentation at the initial stages since it comprises all the essential elements and biosynthetic building blocks necessary for yeast cell propagation [31]. *S. carlsbergensis* RU01 cells were cultured in YM medium with or without 0.1% w/v tannic acid supplementation at 30 °C for 48 h. Subsequently, the cells were fixed with HPF. HPF-TEM showed that the cell wall of *S. carlsbergensis* RU01 contains two outer layers of mannoprotein and an inner β-glucan-chitin layer. During stress, the yeast cells were cultured in tannic acid, leading to significant changes in the cell wall. If one assumes that the yeast cell wall does not change in YM medium (Fig. 1a), it is the tannic acid in the supplemented medium that changes the cell wall composition. In other words, the β-glucan-chitin layer is likely to become thicker, while the mannoprotein layers become thinner (Fig. 1b). The structured side of the yeast is in a near-native state. This analysis revealed dramatic changes in cell wall architecture immediately following supplementation with or without tannic acid. Mongkontanawat et al. [32] observed that the mannoprotein content decreased, while the β-glucan content increased, in the *S. cerevisiae* cell wall. Bzducha-Wróbel et al. [33] found that the β-glucan content increased in the *Candida utilis* cell wall. Moreover, Osumi [34] examined septum formation during cell division after cryofixation by HPF. The α-1,3 and β-1,3 glucans were found in the invaginating nascent septum, and highly branched β-1,6-glucan was later observed on the second septum. However, Mekoue et al. [20] suggested that mannan and β-glucan in yeast cell walls were increased to protect the cells from tannins.

### Effect of Tannic Acid on β-Glucan Production and DCW

**Fig. 2:**
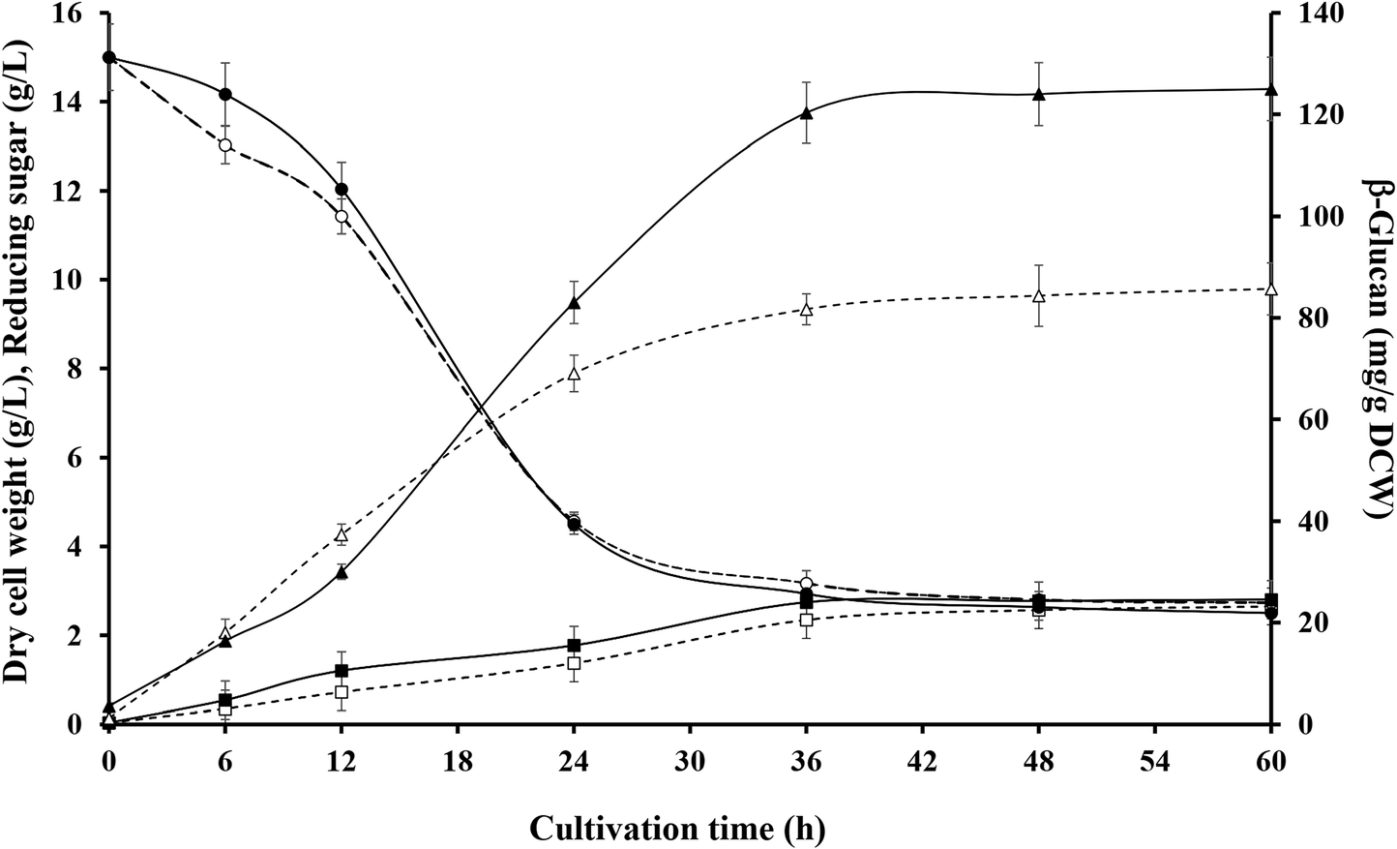
Culture time for production of β-glucan in *Saccharomyces carlsbergensis* RU01 grown in 3% w/v molasses and 0.1% w/v diammonium sulfate (MDS) medium. The slash lines and unfilled symbols indicate MDS medium, and dark lines and solid symbols indicate MDS medium supplemented with 0.1% w/v tannic acid. Symbols: rectangles, dry cell weight (DCW); circles, reducing sugar; and triangles, β-glucan

The culture time for β-glucan production from *S. carlsbergensis* RU01 in MDS medium with or without tannic acid supplementation was investigated. The β-glucan content, DCW, and reducing sugar content were determined as shown in Fig. 2. As the reducing sugar content decreased, a β-glucan content of 11.99% w/w was obtained in the medium supplemented with 0.1% w/v tannic acid, which was higher than that in the medium without tannic acid (8.43% w/w) after 36 h of cultivation. MDS medium supplemented with tannic acid yielded the highest DCW of *S. carlsbergensis* RU01 at 0.28% w/v (Table 1). Yeast cells can protect themselves and maintain internal homeostasis under stress by synthesizing thicker cell walls which increases carbohydrate levels. The cells may elicit a response to tannins by increasing the synthesis of β-glucan, which builds a thicker cell wall [35]. This is consistent with the results of Chotigavin et al. [36], who found that tannic acid at low concentrations enhanced the growth of the yeast *S. carlsbergensis*. Kim et al. [14] found that molasses and corn steep liquor (CSL) increased the cell mass of *S. cerevisiae* JUL3. In addition, Mongkontanawat et al. [37] indicated that YM medium supplemented with additive chemical stress factors, such as EDTA and SDS, increased β-glucan production in *S. cerevisiae* by approximately 7–40%. Phenolic compounds, which are chemical stress factors present in malva nut juice wastewater, were also reported to enhance β-glucan production in *S. cerevisiae* [38]. This indicated that tannins interfered with the outer structure of the yeast cell wall. Tannins had effects on the mannoprotein layers, which caused them to aggregate. Therefore, the structure of the cell wall could be changed by increasing β-glucan production.

**Table 1:** Dry cell weight, β-glucan content, and FTIR range ratios of the polysaccharide, protein, and lipid content of *S. carlsbergensis* RU01 cultured in MDS medium (control) with or without 0.1% w/v tannic acid (TA) supplementation at 36 h

Medium	DCW (%w/v)	β-Glucan content (%w/w)	Band area (%)			Range ratio (1:2:3)
			Range 1 925–1190 cm^−1^ (polysaccharide)	Range 2 1500–1700 cm^−1^ (protein)	Range 3 2800–3000 cm^−1^ (lipid)	
MDS	0.25±0.01^a^	8.43±0.21^a^	11.16	10.57	6.42	1.7:1.6:1.0
MDS + TA	0.28±0.01^b^	11.99±0.04^b^	12.65	9.66	6.67	1.9:1.5:1.0

Data are shown as the mean±SD derived from three replicates. Means within a column followed by a different letter are significantly different (*p*≤ 0.05)

### Scale-up of β-Glucan Production in Batch Fermentation

**Fig. 3:**
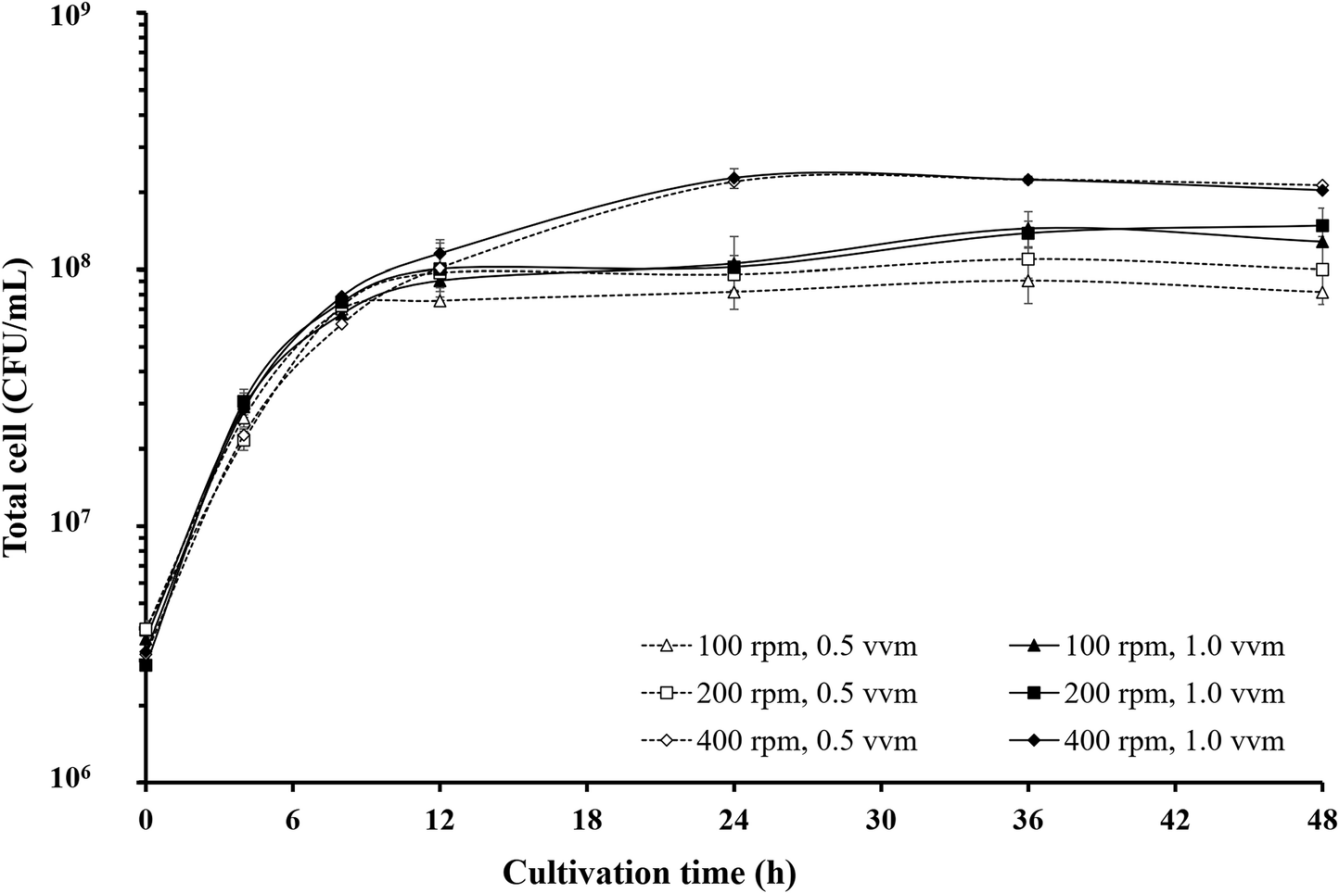
Number of *S. carlsbergensis* RU01 cells in a 5-L stirrer tank reactor under various agitation and aeration conditions

At aeration rates of 0.5 and 1.0 vvm, similar amounts of cells were obtained at the same agitation rate; the aeration rate did not affect cell growth, while agitation at 100, 200, and 400 rpm affected the total yeast cell count, as shown in Fig. 3. The cell count was proportional to the agitation rate, and 400 rpm yielded the highest amount of yeast cells. Therefore, the optimum agitation rate for cell growth was 400 rpm with aeration rates of 0.5 and 1.0 vvm. The results in Table 2 show that the β-glucan levels at 36 h were 14.61 and 17.08% w/w, whereas at 48 h, the β-glucan levels were 16.19 and 14.95% w/w. Based on the results, agitation at 400 rpm with aeration at 1.0 vvm afforded the highest β-glucan content at 36 h. It was suggested that the oxygen concentration influenced the β-glucan content. According to Baez and Shiloach [39], oxygen content affects bacteria and yeast growth by accelerating cell division, but it affects antioxidants and causes deterioration of the repair pathway itself. The cells may have thinner cell walls and decreased amounts of β-glucan.

**Table 2:** β-Glucan content of *S. carlsbergensis* RU01 cultured in a 5-L stirred tank reactor in MDS medium supplemented with 0.1% w/v tannic acid at 36 and 48 h

Agitation/aeration rate	β-Glucan content (%w/w)			
	36 h		48 h	
	0.5 vvm	1.0 vvm	0.5 vvm	1.0 vvm
100 rpm	4.37±0.52	5.44±0.45	4.80±0.48	5.90±0.37
200 rpm	7.87±0.32	10.10±0.60	9.67±0.81	12.41±0.61
400 rpm	14.61±0.42	17.08±0.55	16.19±0.61	14.95±0.43

Data are shown as the mean±SD derived from three replicates

The *S. carlsbergensis* RU01 DCW, β-glucan content, and fold change in β-glucan yield are shown in Table 3. The relation between growth and fermentation rates was first established. After 36 h of cultivation in a shake flask (SF), approximately 119.89 mg/g DCW was obtained. Batch fermentation contributed to an increase in β-glucan production of 172.26 mg/g DCW. Thus, the efficacy of batch fermentation in β-glucan production was higher than that of batch culture. In addition, the β-glucan yield coefficient (Yp/s) for the cultivation mode of *S. carlsbergensis* RU01 was defined as the amount of β-glucan produced per unit of reducing sugar consumed at 36 h. The results showed that batch fermentation and SF cultivation consumed 0.059 and 0.028 g/g reducing sugars, respectively.

**Table 3:** Dry cell weight and yield and fold change of β-glucan in *S. carlsbergensis* RU01 cultured in a shake flask or 5-L stirred tank reactor in MDS medium supplemented with 0.1% w/v tannic acid for 36 h

Cultivation mode	DCW (g/L)	β-Glucan content (mg/g DCW)	(Y_p/s_) (g/g reducing sugar consumed)	Fold change of β-glucan
Shake flask	2.81±0.11^a^	119.89±0.38^a^	0.028	1.0
Stirred tank reactor	4.07±0.16^b^	172.26±9.05^b^	0.059	1.4

The data are shown as the mean±SD derived from three replicates. Means within a column followed by a different letter are significantly different (*p*≤0.05)

### Composition of β-Glucan from *S. carlsbergensis* RU01 by FTIR Analysis

The FTIR spectrum was used to study three main regions, corresponding to polysaccharides (925–1190 cm^−1^), proteins (1500–1700 cm^−1^), and lipids (2800–3000 cm^−1^), in yeast cells [31, 40]. Curve fitting and band area determination have been previously employed to analyze yeast cell walls [41, 42]. FTIR analysis of the extracted β-glucan from *S. carlsbergensis* RU01 was performed. The polysaccharide, protein, and lipid levels of the glucan were calculated from the band area (%). The alkaline extract of *S. carlsbergensis* RU01 cell walls was designated β-glucan because it contained this polysaccharide as the main component, mixed with proteins and lipids (Table 1). The extracted β-glucan of *S. carlsbergensis* RU01, which was cultured in MDS medium supplemented with 0.1% w/v tannic acid, showed a higher ratio (1.9:1.5:1.0) and %polysaccharide than the control. The results showed that the polysaccharide content was higher than that in the control, while the protein content was slightly lower than that in the control. It was suggested that tannic acid enhanced the β-glucan content and decreased the protein content in the cell wall of *S. carlsbergensis* RU01.

## Conclusion

β-Glucan production in *S. carlsbergensis* RU01 was enhanced by tannic acid. The cell wall of *S. carlsbergensis* RU01 became thick, and β-glucan production increased significantly. The β-glucan content was enhanced 42.23% by tannic acid and 1.4-fold upon changing the mode of cultivation. The extracted β-glucans were mainly polysaccharides, mixed with proteins and lipids. In addition, β-glucans from brewer’s yeast can be used as a prebiotic and functional foods for human health and in animal feed.
